# Supplementary material for: CHAI: consensus clustering through similarity matrix integration for cell-type identification
Source: Brief Bioinform. 2024 Aug 29;25(5):bbae411. doi: 10.1093/bib/bbae411 (PMC11359802; doi:10.1093/bib/bbae411)
Supplement: BIB_CHAI_Supp_bbae411 [file bib_chai_supp_bbae411.pdf]

CHAI: Consensus Clustering Through Similarity Matrix Integration for Cell-Type Identification

Musaddiq K Lodi
Integrative Life Sciences
Virginia Commonwealth University
Richmond, VA 23284, USA
lodimk2@vcu.edu

Muzammil K Lodi
Department of Computer Science
Virginia Commonwealth University
Richmond, VA 23284, USA
lodimk4@vcu.edu

Kezie Osei
Center for Biological Data Science
Virginia Commonwealth University
Richmond, VA 23284, USA
oseika@vcu.edu

Vaishnavi Ranganathan
School of Computer Science
Carnegie Mellon University
Pittsburgh, PA 15213, USA
vrangana@andrew.cmu.edu

Priscilla Hwang
Department of Biomedical Engineering
Virginia Commonwealth University
Richmond, VA 23284, USA
hwangp2@vcu.edu

Preetam Ghosh
Department of Computer Science
Virginia Commonwealth University
Richmond, VA 23284, USA
pghosh@vcu.edu

1 Supplementary Materials

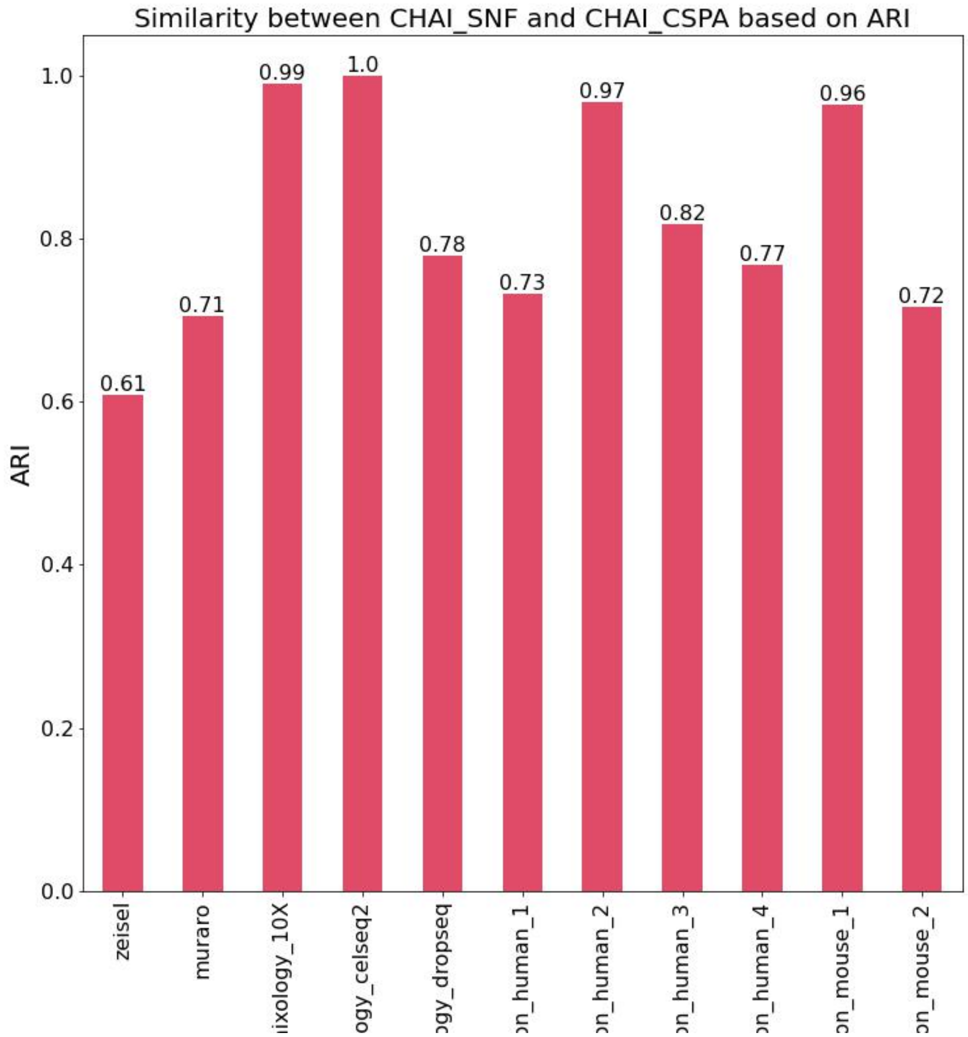

Figure S1: ARI Comparison between CHAI-SNF and CHAI-AvgSim by ARI. Despite differences in the ground truth evaluation, the similarity between clustering assignments by CHAI-AvgSim and CHAI-SNF is quite similar

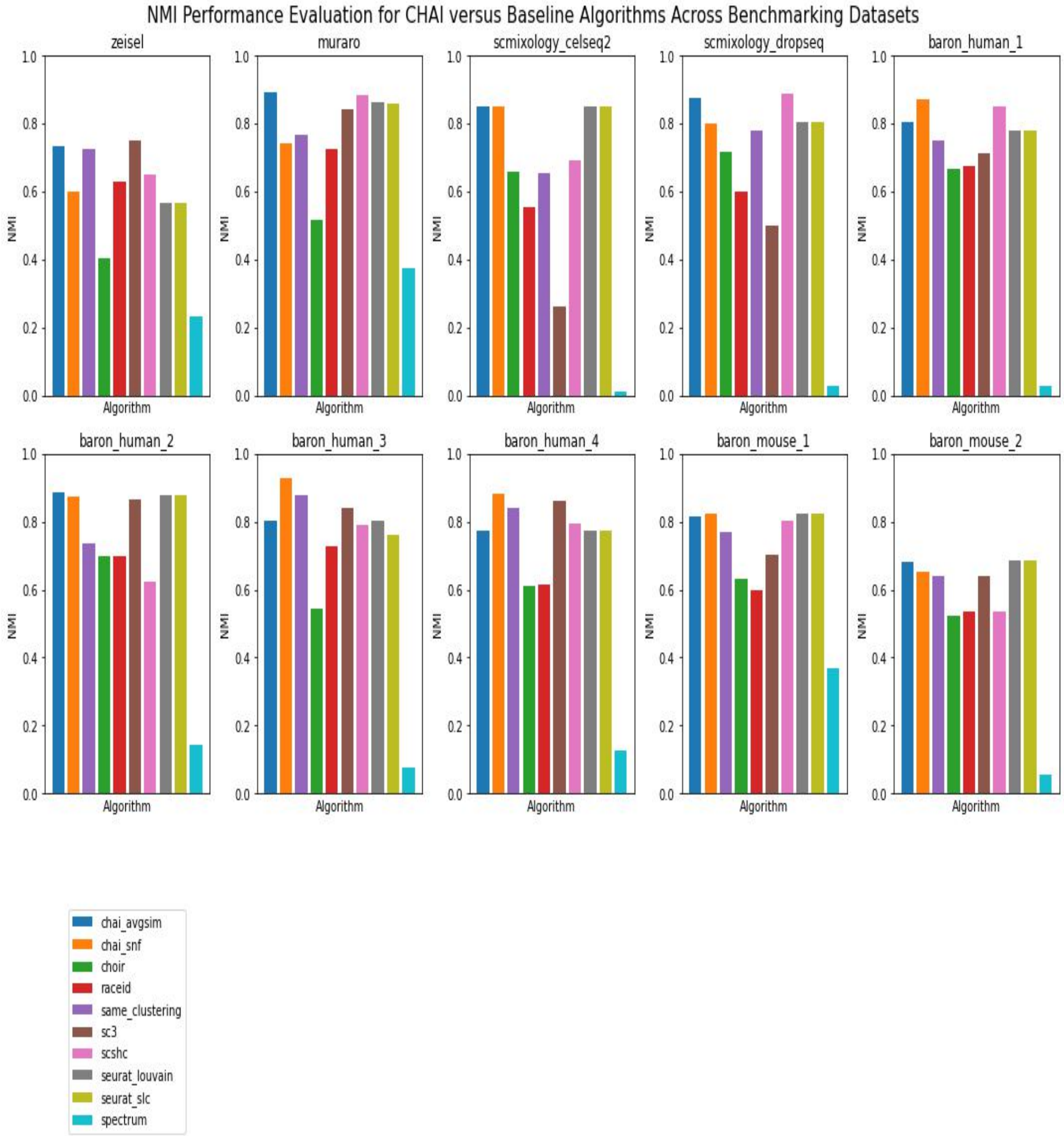

Figure S2: NMI Evaluation for Benchmarking Datasets

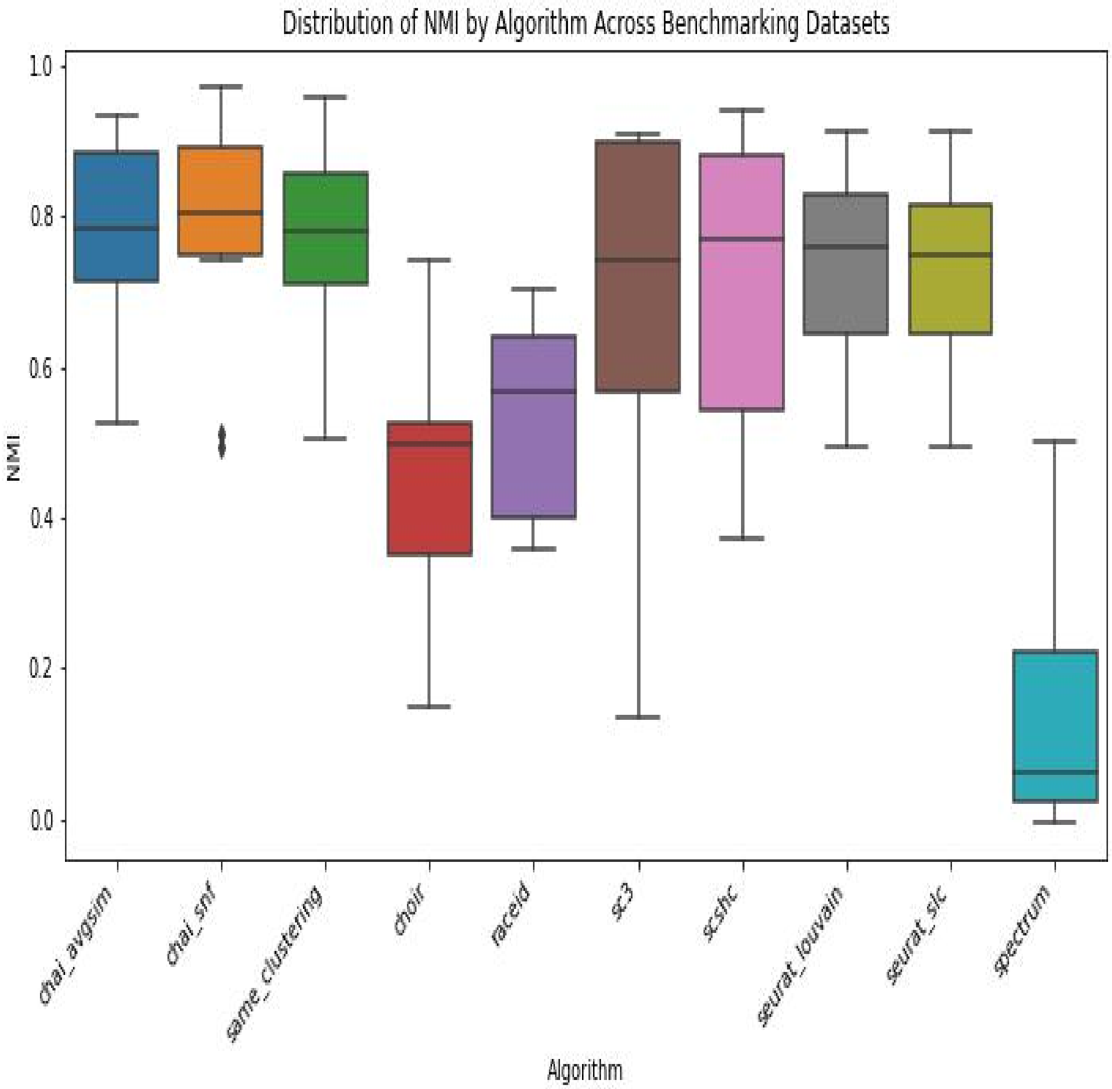

Figure S3: NMI Distribution for Benchmarking Datasets

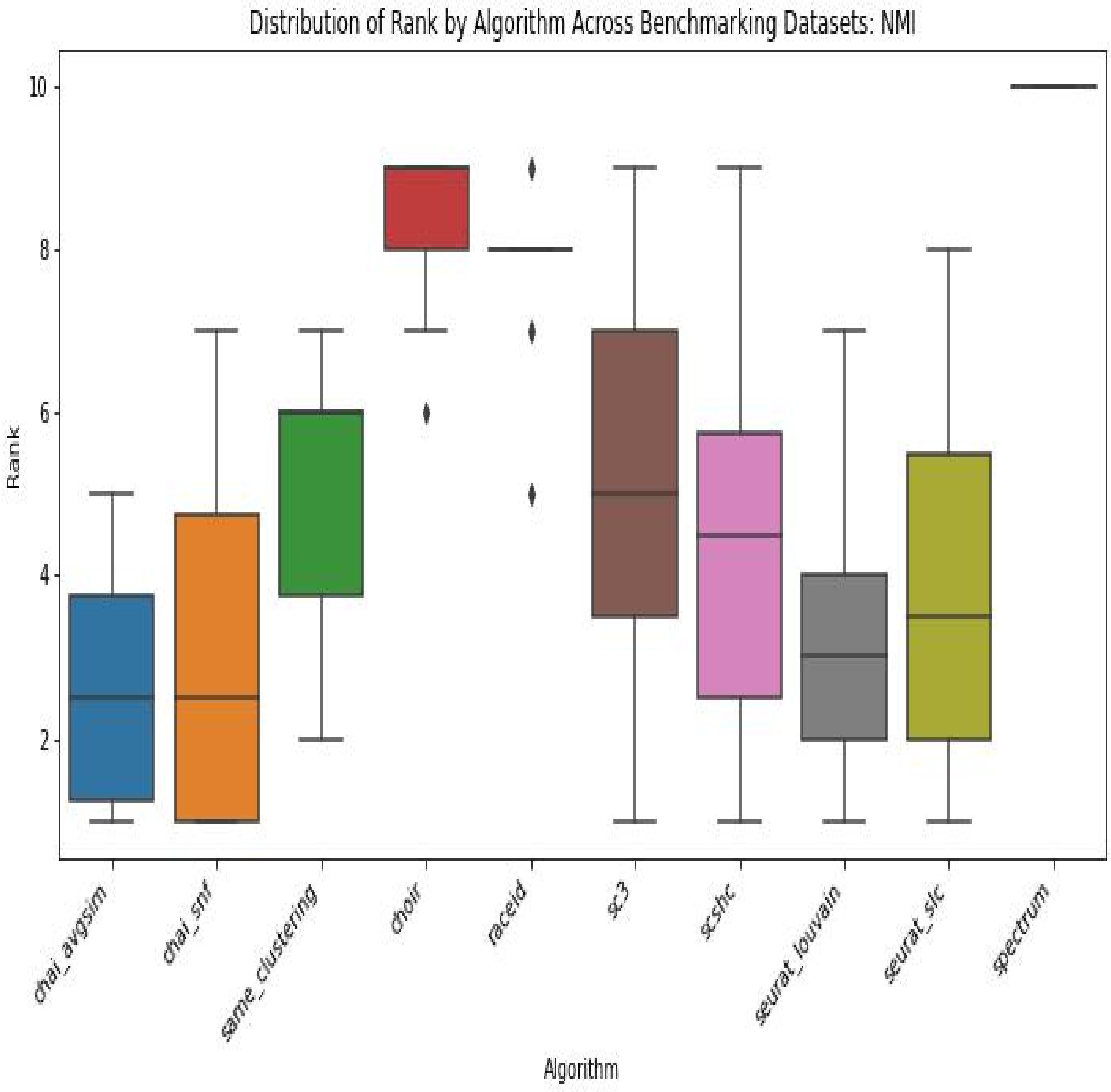

Figure S4: NMI Based Ranking Distribution for Benchmarking Datasets

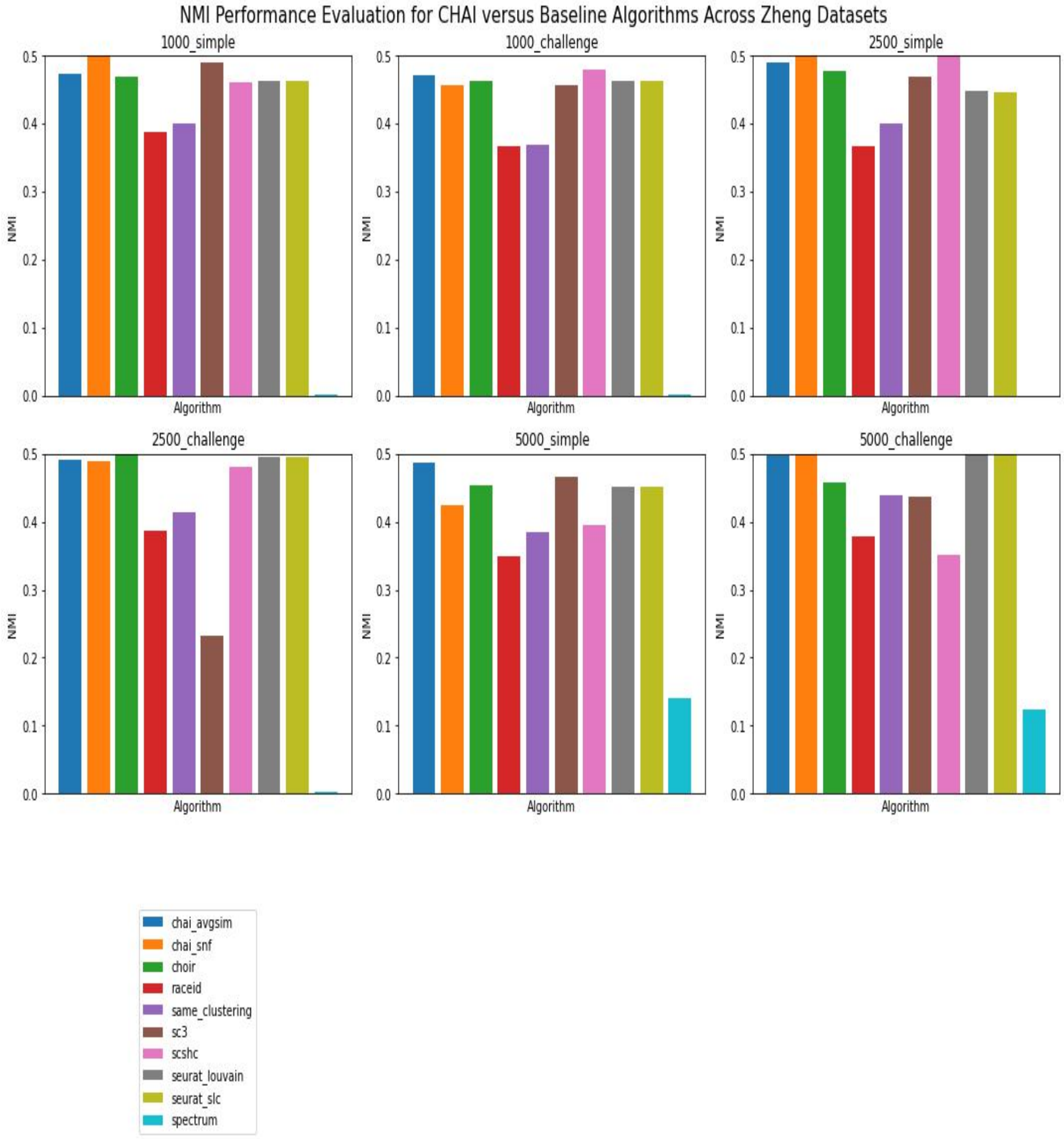

Figure S5: NMI Evaluation for Zheng Datasets

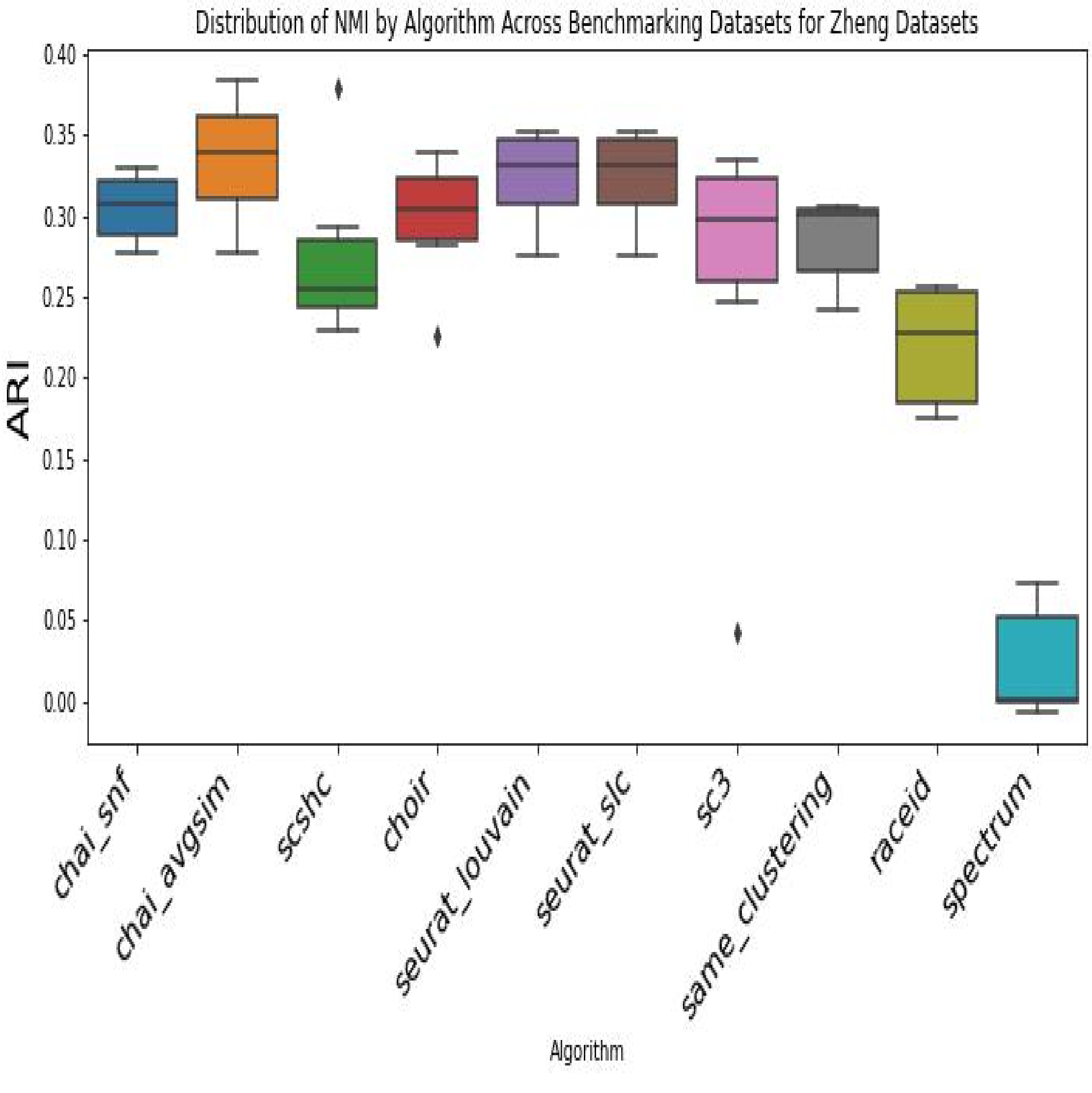

Figure S6: NMI Distribution for Zheng Datasets

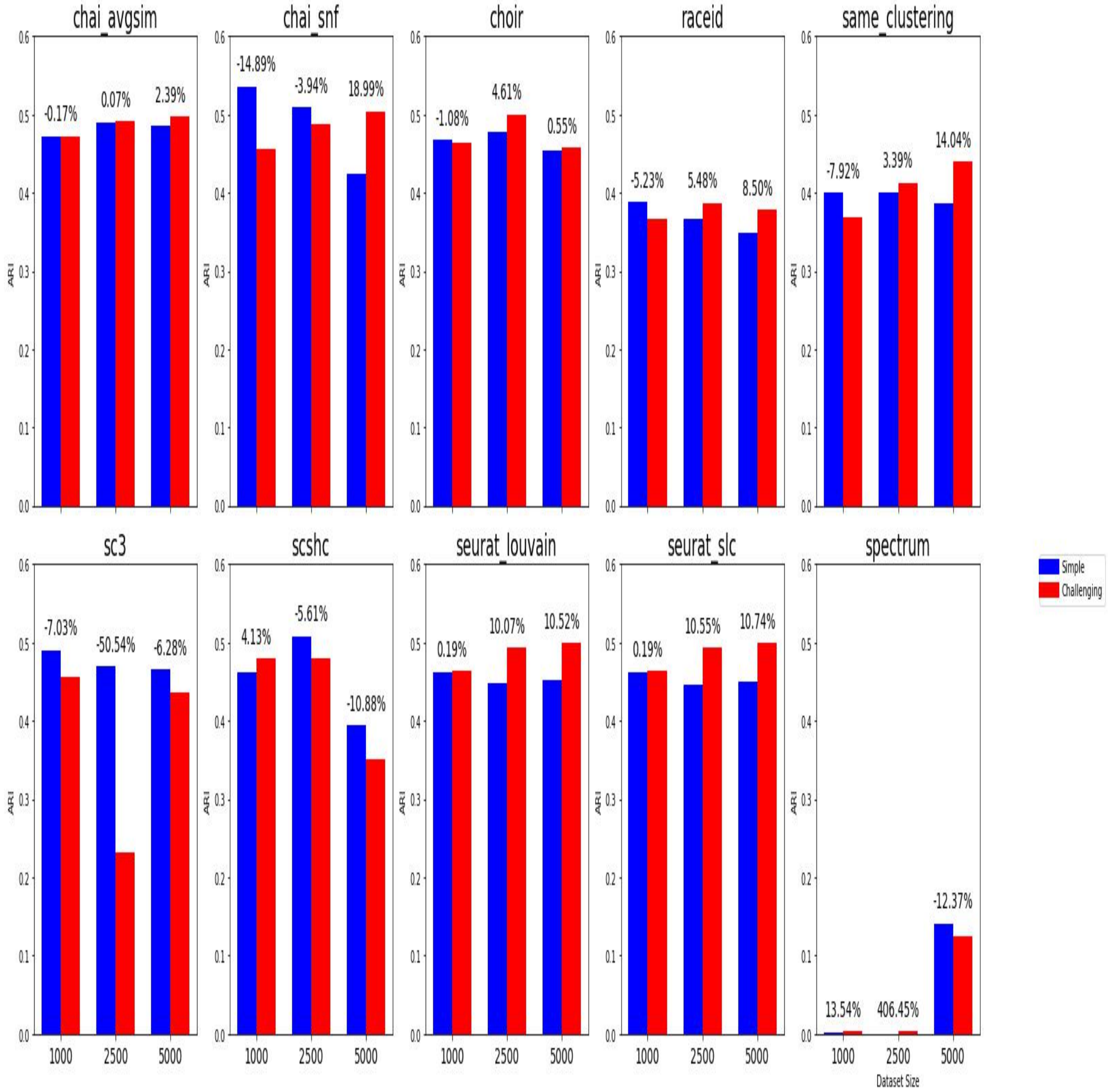

Figure S7: NMI Difference Between Simple and Challenging Datasets for Zheng Datasets

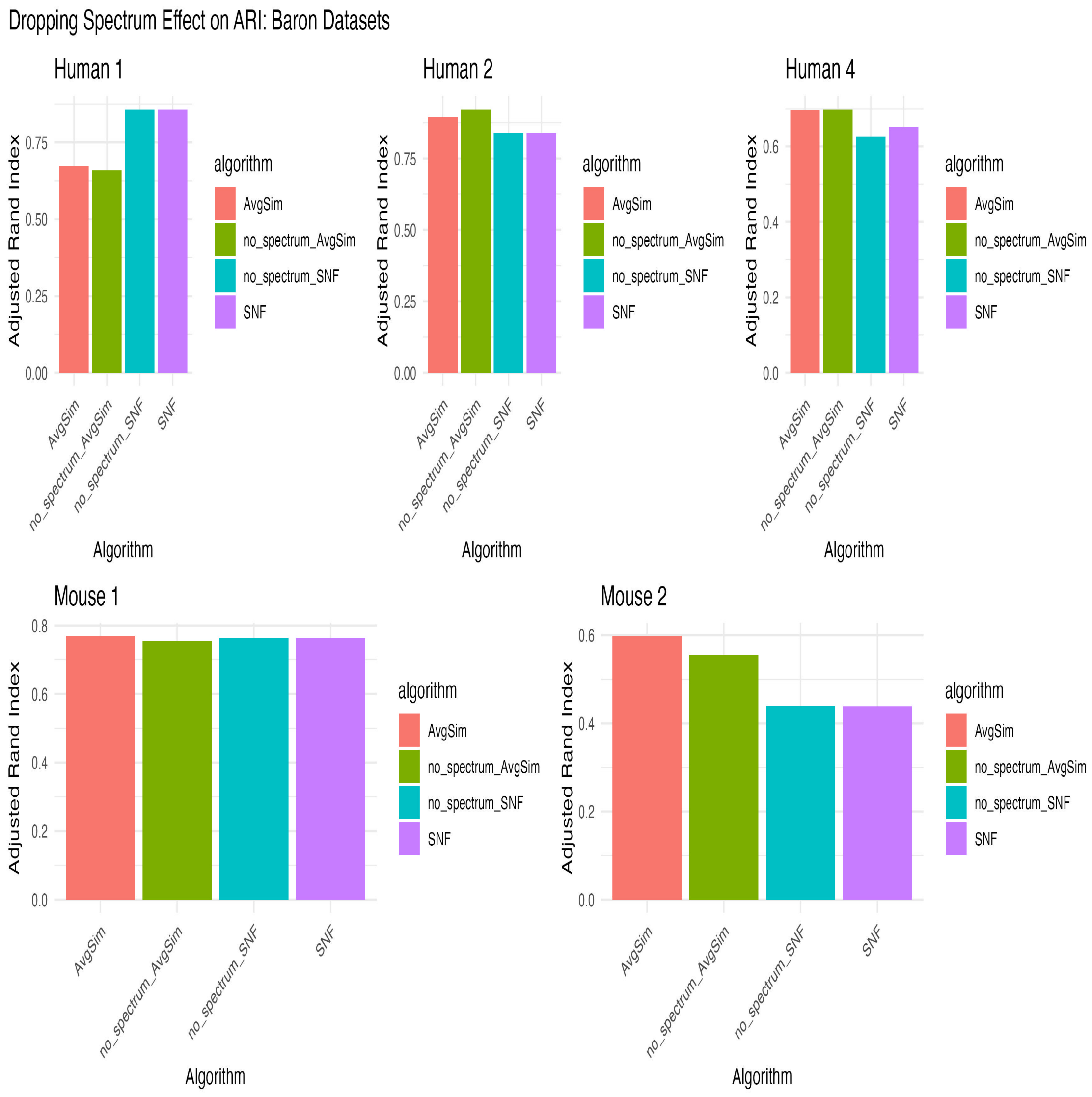

Figure S8: ARI Evaluation when Spectrum is dropped from AvgSim and SNF
